# Early Recognition of Patients with Neuronopathic Forms of Mucopolysaccharidosis Type II in Clinical Practice

**DOI:** 10.3390/medsci14060622

**Published:** 2026-09-30

**Authors:** Natalia V. Buchinskaia, Ekaterina Yu. Zakharova, Anastasia O. Vechkasova, Irina A. Chikova, Nato D. Vashakmadze, Leyla S. Namazova-Baranova, Dmitry O. Ivanov, Sergei I. Kutsev, Mikhail M. Kostik

**Affiliations:** 1 Department of Hospital Pediatry, Saint-Petersburg State Pediatric Medical University, Litovskaya, 2, Saint Petersburg 194100, Russia; nbuchinskaia@gmail.com (N.V.B.); irinachikova@gmail.com (I.A.C.); spb@gpmu.org (D.O.I.); 2 Department of Clinical Genetics, Saint-Petersburg State Medical Diagnostic Center (Genetic Medical Center), Saint Petersburg 194044, Russia; vechkasova.nastia@mail.ru; 3 Molecular and Genetic Diagnostics, Federal State Budgetary Scientific Institution, Research Center for Medical Genetics, Moscow 115522, Russia; doctor.zakharova@gmail.com (E.Y.Z.); kutsev@mail.ru (S.I.K.); 4 Institution of the Maternity and Childhood, Pirogov Russian National Research Medical University, Moscow 117513, Russia; nato-nato@yandex.ru (N.D.V.); leyla.s.namazova@gmail.com (L.S.N.-B.); 5 Department of the Orphan Diseases, Research Institute of Pediatrics and Children’s Health in Petrovsky National Research Centre of Surgery, Moscow 119991, Russia

**Keywords:** mucopolysaccharidosis type II, genotype-phenotype, comparative analysis, early recognition, neuronopathic and non-neuronopathic forms, registry, Russian Federation

## Abstract

***Background*:** Mucopolysaccharidosis type II (MPS II) is a progressive, rare, X-linked inherited disease with multi-organ involvement and a restricted life expectancy. The disease has two main forms: neuronopathic (severe) and non-neuronopathic (attenuated). ***Aim*:** To compare the two main forms of MPS II to identify signs for early recognition of severe disease forms in daily practice. ***Methods*:** In this retrospective cohort study, clinical and laboratory data, as well as enzyme replacement therapy data, for approximately 162 patients were extracted and analyzed from the Russian MPS II registry. Patients with insufficient clinical information (23 patients) and children under 5 years of age (15 patients) were excluded. We compared patients with MPS II neuronopathic (*n* = 82, 66.1%) and non-neuronopathic forms (*n* = 42, 33.9%) based on the presence of cognitive deficits after age 6 years and on remaining committed to the phenotype throughout follow-up. ***Results*:** The patients with both forms had the following symptoms with similar frequency: Hurler phenotype, hernias, hepatosplenomegaly, short neck, orthopedic problems: spinal pathology (curvature), chest deformity, joint stiffness, deformity of the hand, hand contractures, wrist joint deformity and contractures, elbow joint deformity and contractures, shoulder and knee deformity and contractures, hip contractures, cardiomyopathy, obstructive respiratory tract diseases, noisy breathing, hearing loss, carpal tunnel syndrome. Highly specific symptoms (frequency of occurrence above 80%) for the diagnosis of the neuronopathic form of MPS II were: delayed intellectual disability at the age of 1–3 years, epilepsy, and the presence of gross rearrangements in the *IDS* gene. Highly sensitive symptoms (above 80%) were: psychomotor development delay of up to 1 year of age and delayed intellectual disability at the ages of 1–3 years, heart damage, and myxomatous degeneration of the valves (79.5%). A symptom with high sensitivity and specificity is delayed mental and speech development between the ages of one and three, with respective sensitivities and specificities of 94.8 and 86.5. ***Conclusions*:** Identifying symptoms characteristic of neuronopathic forms of MPS is important not only for determining the clinical form of the disease but also for developing various approaches to treating such patients. Early diagnosis of severe forms of MPS II will make it possible to use currently approved methods of intraventricular enzyme replacement therapy, or primarily use of ERT capable of crossing the brain-blood barrier which could improve the prognosis for such patients. Symptoms that predict the risk of developing the neuronopathic form can be used in clinical practice.

## 1. Introduction

Mucopolysaccharidosis type II (MPS II) is a rare inherited X-linked disease with pathogenic variants of the *IDS* gene, reduced activity of iduronate-2-sulfatase (IDS), and accumulation of glycosaminoglycans (GAGs) in lysosomes [1]. It is characterized by multi-organ involvement, a progressive course, and restricted life expectancy.

In 2/3 of cases, the accumulation of glycosaminoglycans leads to the development of a severe or neuronopathic form of MPS II with central nervous system damage and the development of cognitive deficits [2]. The mild form of MPS II is characterized by a later onset of symptoms, absence of hydrocephalus syndrome, epilepsy, and significant cognitive deficits. Long-term follow-up of patients with mild forms of MPS II shows possible involvement of the central nervous system in the late stages of the disease [3]. The average age of the first symptoms is 1.5 and 4.3 years for the neuronopathic/non-neuronopathic forms, respectively [4] or 1.5 and 2.0 according to later data in 2023 [5].

The early differential diagnosis between the two clinical forms of type II MPS is complicated, and the aim of our study was to early identify predictors of a severe disease phenotype to help specialists working with orphan diseases to early identify a group of children at high risk of developing the neuronopathic form and start the treatment with the drugs capable of crossing the blood–brain barrier in order to avoid or diminish cognitive deficits in this group of patients. The known literature has not previously formulated predictors of the development of the neuronopathic form of the disease, except for individual publications describing differences between the forms of MPS type II in patients older than 6–10 years [5,6].

According to the literature, the exact differentiation of MPS into neuronopathic and non-neuronopathic forms at an early age is challenging, especially in the absence of significant involvement of the central nervous system. According to various researchers, it is most reliable to consider the presence of a non-neuronopathic form only at the age of 6 years [6] or even after 10 years [5]. Therefore, a patient with a presumably mild form under the age of 6 years may develop into a neuronopathic form due to slow progressive involvement of the central nervous system.

Early differentiation of the two types of MPS II is crucial, since this directly affects the approaches to therapy: in neuronopathic forms, it is possible to use not only intravenous, but also intraventricular/intrathecal therapy, as well as gene therapy to decrease central nervous system (CNS) damage [7,8,9]. Since 2008, intravenous enzyme replacement therapy (ERT) with the drugs idursulfase and idursulfase beta has been approved and available worldwide and in Russia. Numerous studies have shown the safety and effectiveness of these therapies for the treatment of somatic manifestations of MPS II [10,11,12]. The limitation of intravenous ERT is the inability of the drug to penetrate the blood–brain barrier. Therefore, new approaches to ERT (modification of the enzyme delivery method) and gene therapy of neuronopathic forms of MPS II are currently being developed [2,12] and have been put into practice in recent years [13,14].

The intracerebroventricular administration of idursulfase beta, according to a 5-year follow-up of patients with the neuronopathic form of MPS II, has shown the safety and effectiveness of such therapy. There was an improvement in neurocognitive development, which correlated with a decrease in the excretion of heparan sulfate in the cerebrospinal fluid. The best effect was observed in the group of children starting therapy at the age of 3 years and younger [8,9].

The identification of symptoms characteristic of the severe form of MPS and the search for predictors of severe forms of MPS II are important not only to establish the form of the disease clinically, but also in connection with different approaches to the treatment of such patients.

Early access to therapy improves the prognosis of the disease and reduces the degree of progression of MPS II. The initiation of ERT before the age of 3 years has shown the greatest effectiveness on the somatic condition of patients not only in non-neuronopathic forms, but also in neuronopathic ones [15].

Our study aimed to find the early predictors of neuronopathic forms of MPS II.

## 2. Materials and Methods

This retrospective study cohort included the clinical, epidemiological, instrumental, and laboratory data from about 162 patients from the Russian registry of patients with MPS II. The first patient was included in 2008; the last data were downloaded in November 2024. Participation in the registry was voluntary: The patient or his legal representative signed an informed consent to include the data. Data were collected and analyzed using the Quinta CRM (31-131 Kraków, Poland) platform (certificate of state registration of computer programs # 2016615129 for Quinta: Universal software for remote collection, processing, and management of geographically distributed clinical and epidemiological data; copyright holder—Aston Consulting) in partnership with Aston Consulting.

**Figure 1:**
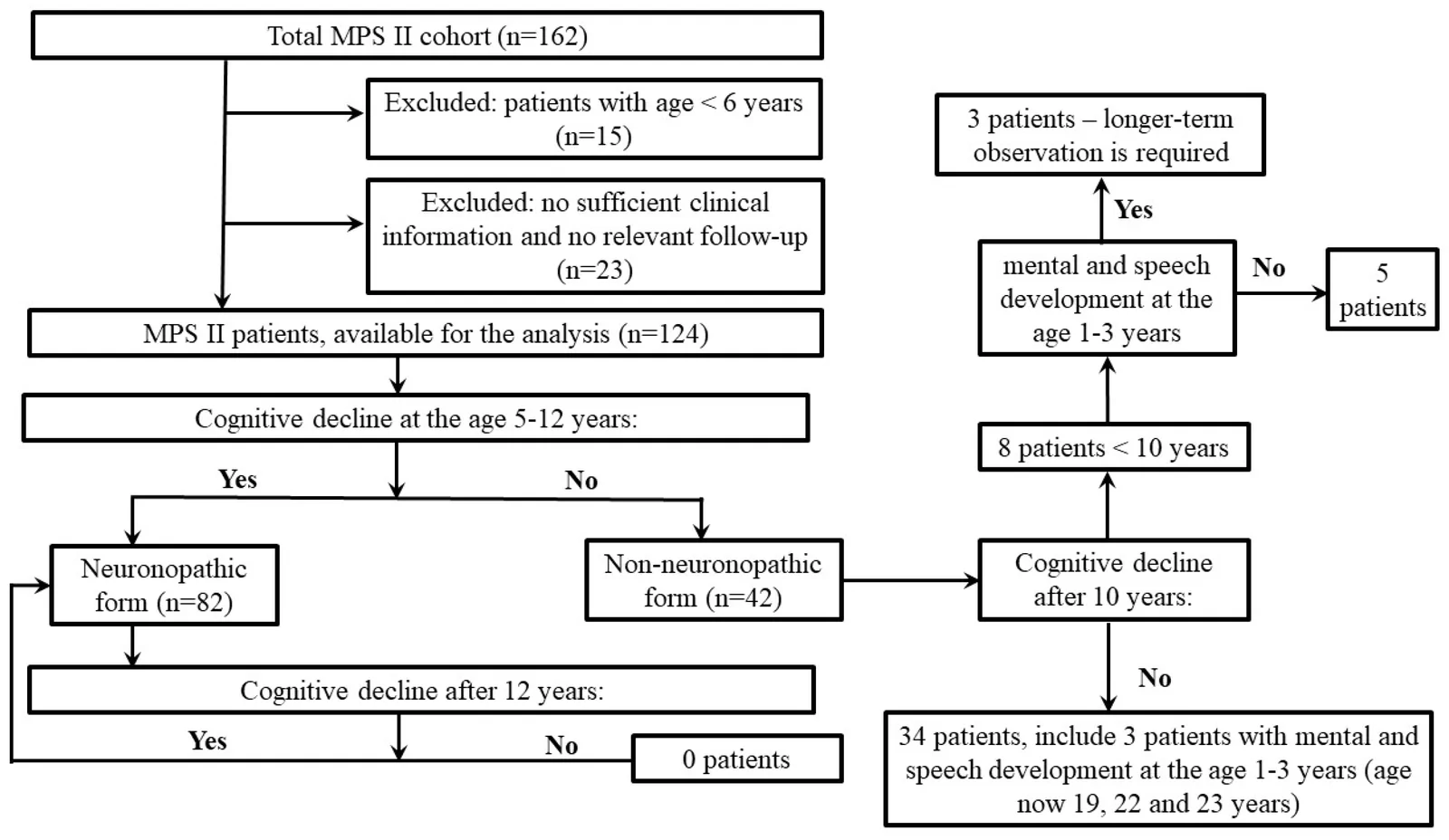
Flow chart of the selection of the patients with MPS II in the study.

To conduct the study, information about 162 patients was extracted from the Russian registry of MPS II patients. Patients with insufficient clinical information to determine the phenotype (23 patients), as well as children under 6 years of age (15 patients), for whom it is difficult to establish the phenotype of the disease, were excluded from further study. The remaining 124 patients were divided into 2 groups, depending on the presence of cognitive deficits over the age of 6 years and remaining committed to the phenotype throughout follow-up in dynamics: neuronopathic (severe) and non-neuronopathic (mild) based on previous studies [2,16]. The principle of selection of the patients in two groups is shown in Figure 1.

The study was approved by the Ethics Committee of Saint-Petersburg State Pediatric Medical University (protocol #1 from 19 January 2009).

In all cases, the diagnosis of MPS II was confirmed by high urine excretion of glycosaminoglycans (dermatan sulfate and heparan sulfate) and enzymatic iduronate sulfatase deficiency in leukocytes, plasma, or dried blood spots. Data on enzyme activity and molecular genetic studies in this patient were unavailable. Multiple possible sulphatase deficiency was excluded by measuring another sulphatase in lysosomal disease screening.

### 2.1. Assessments and Outcomes

We extracted the following patients’ information for the assessment: demographics: age, sex, residence, family history of MPS II, age at first symptom(s), age at diagnosis, and time to diagnosis. Clinical features related to the disease: The main clinical symptoms were associated with the last data assessment, and some clinical signs were assessed twice in the first and last observations of the study. Laboratory data: The genetic analysis data (sequencing by Sanger) showed significant changes in the *IDS* gene, including deletions, recombinant events involving *IDS-IDS2*, and missense mutations leading to a severe phenotype of MPS II. Treatment: The number of patients treated with enzyme replacement therapy (ERT), ERT onset age and duration, ERT delay.

### 2.2. Outcomes

The main outcomes were cognitive impairment, alive or dead. The observation time was calculated using the date of death (for dead patients) or the date of the last available observation included in the registry.

We compared patients who had neuronopathic (*n* = 82, 66.1%) and non-neuronopathic forms (*n* = 42, 33.9%) of MPS II from 124 patients.

The change in the degree of cognitive deficit over time was assessed retrospectively at the age of 1 year, 1–3 years, 5–12 years, as well as over the age of 12 years. Other symptoms of central nervous system damage, such as hydrocephalus, epilepsy, apnea, swallowing disorder, and independent walking, were evaluated.

### 2.3. Statistical Analysis

We utilized the STATISTICA software package, version 10.0 (StatSoft Inc., St. Tulsa, OK, USA). Numerical indicators were presented with the median and interquartile range (IQR; 25th and 75th percentiles), and categorical variables were presented with absolute numbers and the percentages (%). We used the Pearson χ^2^ to compare the independent categorical variables and the Mann–Whitney test for independent numerical variables. Each predictor associated with development of the neuronopathic form was assessed by analyzing the sensitivity and specificity. We identified independent predictors of neuronopathic form development as the event of interest using binary logistic regression by including qualitative indicators related to the dependent variable in the analysis. A prognostic model for the risk of ascertained outcome (neuronopathic form development) was constructed using multivariate logistic regression. The independent variables were selected using stepwise direct selection with the Wald statistic as an exclusion criterion. All significant predictors were included in the following multiple regression analysis. Differences were considered statistically significant if the *p* value was less than 0.05.

## 3. Results

The study included 124 patients over 5 years of age with a diagnosis of MPS II: 82 patients with the neuronopathic (severe) form (66.1%) and 42 patients with the non-neuronopathic (mild) form (33.9%).

Patients with MPS II neuronopathic variants had an earlier age of onset of symptoms and an earlier age of diagnosis. The median age of the first symptoms was 1 year vs. 3 years, and the median age of diagnosis was 3 years vs. 6 years for the neuronopathic and non-neuronopathic forms, respectively.

### 3.1. Dynamic Assessment of Neurocognitive Development

In patients with neuronopathic forms, there was an increase in the number of symptoms of CNS damage and cognitive impairment with increasing age of patients compared with non-neuronopathic forms. A detailed description of the dynamics of psychomotor, speech, and cognitive development in MPS II patients is presented in Table 1.

### 3.2. Clinical Characteristics of Patients with Neuronopathic and Non-Neuronopathic Forms of MPS II

The patients with both forms had the following symptoms with similar frequency: Hurler phenotype, hernias, hepatosplenomegaly, short neck, orthopedic features: spinal pathology (curvature), chest deformity, joint stiffness, deformity of the hand, hand contractures, wrist joint deformity and contractures, elbow joint deformity and contractures, shoulder and knee deformity and contractures, hip contractures, cardiomyopathy, obstructive respiratory tract diseases, noisy breathing, tracheostomy, hearing loss, carpal tunnel syndrome.

#### Joint Involvement

The frequency of patients with joints stiffness was similar between groups: 78/81, 96.3%, and 35/37, 94.6% for the neuronopathic and non-neuronopathic forms. The main differences in the frequency of joint involvement were obtained for the ankle, knees, and feet joints, predominantly as deformities, in neuronopathic forms. Pain during joint movements was a rare complaint of patients with MPS II in both groups, except that knee pain reached a difference of 14% (8/57) in patients with the neuronopathic form compared to 0/31 (0%) in patients with the non-neuronopathic form (*p* = 0.029). Data are presented in Table 2.

### 3.3. Molecular Differences Between Two Forms

Molecular genetic studies were performed in 101/124 patients: neuronopathic/non-neuronopathic—63/101 (62.4%)/38/101 (37.6%). Point mutations occurred in 88/101 patients, and 13/101 had gross rearrangements. The most common point mutations (85/88 patients) were found in exon 3 of the *IDS* gene in 18 (21.2%) patients (neuronopathic/non-neuronopathic—11/82 (13.4%)/7/42 (16.7%)) and in exon 9 (18.8%) in 16 patients (neuronopathic/non-neuronopathic—12/82 (14.6%)/4/42 (9.5%)), respectively. The missense variants were found in 57/88 (64.8%) patients (neuronopathic/non-neuronopathic forms 32/63 (50.8%) vs. 25/38 (65.8%); *p* = 0.141), nonsense variants in 8/88 (9.1%) patients (neuronopathic/non-neuronopathic—4/63 (6.3%) vs. 4/38 (10.5%); *p* = 0.452), splicing substitutes 11/88 (12.5%) (neuronopathic/non-neuronopathic—4/63 (6.3%) vs. 7/38 (18.4%); *p* = 0.060), frameshift mutations in 12/88 (13.6%) patients (neuronopathic/non-neuronopathic—11/63 (17.5%) vs. 1/38 (2.6%); *p* = 0.026). The significant difference in the distribution of missense variants in neuronopathic and non–neuronopathic forms was observed (*p* = 0.04). Pathogenic variants occurred in 68/101 patients (67.3%), including neuronopathic/non-neuronopathic forms 44/63 (69.8%) vs. 24/38 (63.2%; *p* = 0.488), probably pathogenic in 24/101 (23.8%) patients (neuronopathic/non-neuronopathic forms—17/63 (27.0%) vs. 7/38 (18.4%); *p* = 0.328), variants of unknown significance (VUS) in 9/101 (8.9%) patients (neuronopathic/non-neuronopathic forms—2/63 (3.2%) vs. 7/38 (18.4%); *p* = 0.010). Characteristics of the identified variants: severe variants (pathogenic missense mutations and gross rearrangements of the *IDS* gene) were detected in 46/101 patients (45.5%): 30/46 (65.2%) in the neuronopathic form and 16/46 (34.8%) in the non-neuronopathic form (*p* = 0.004). Gross rearrangements were observed in 13/101 patients, including neuronopathic/non-neuronopathic forms 12/63 (19.0%) vs. 1/38 (2.6%; *p* = 0.018).

### 3.4. Predictors of the Following Development of MPS Type II Neuronopathic Form

The sensitivity, specificity, and odds ratio of symptoms with significant differences in neuronopathic and non-neuronopathic forms were analyzed.

The psychomotor development delay up to 1 year, delayed mental and speech development at the age of 1–3 years, swallowing disorders, independent walking failure at any age and foot deformity showed the highest specificity, and delayed mental and speech development (1–3 years), hydrocephalus, cardiac disorders (hypertrophic cardiomyopathy and myxomatous valve disease) showed the highest sensitivity in the discrimination between the two forms. Taking into account that the mean age of the neuronopathic form diagnosis is 3 (2; 5) years, the main practical meaning had the following predictors: psychomotor development delay (up to 1 year), delayed mental and speech development (1–3 years), hydrocephalus, epilepsy, and heart involvement. The results are presented in Table 3, and the most meaningful predictors are bolded.

Multivariate regression analysis (Table 4) identified the following predictors of the following development of neuronopathic forms of MPS type II (r^2^ = 0.66, *p* < 0.0000001): psychomotor development delay up to 1 year (*p* = 0.01), delayed mental and speech development at the age of 1–3 years (*p* = 0.0000001), cardiac disorders (*p* = 0.058).

## 4. Outcomes

Adherence to therapy was significantly (*p* = 0.0018) higher in patients with neuronopathic forms and amounted to 96.1% vs. 76.2% in patients with the non-neuronopathic form of MPS II. Among the deceased, 13/82 (15.7%) patients had a severe form of the disease, and 5/41 (12.2%) had an attenuated form; the vital status of one patient is unknown. The most common causes of death were cardiovascular failure in five (two of them had the neuronopathic form, 2/82, 2.4%) out of 18 cases (27.8%), and respiratory failure in five cases (27.8%), three of whom had neuronopathic form (3/82, 3.7%). Pneumonia or generalized viral infection was reported as a cause of death in three out of 18 patients (16.7%)—two with neuronopathic form (2/82, 2.4%), and cardiorespiratory failure led to death in two neuronopathic patients (11.1%, 2/82, 2.4% neuronopathic patients). The progression of MPS II was indicated as the cause of death for two neuronopathic patients (11.1%, 2/82, 2.4% neuronopathic patients). Acute or chronic respiratory failure, either alone or in combination with other causes, was present in nine (five with neuronopathic form, 5/82, 6.1%; four with non-neuronopathic form, 4/42, 9.5% with) out of 18 patients (50%), and in three patients the cause of death was not indicated.

## 5. Discussion

The majority of the previously published papers did not show a strong association between genotype and phenotype in MPS II patients [17], as well as between glycosaminoglycan urine excretion and phenotype severity [18].

In a recent Chinese study [6], the association between gross rearrangements and severe phenotype was first shown, followed by the data of the Hunter Outcome Survey (HOS) registry [19]. Missense mutations and gross rearrangements of *IDS/IDSP1* were the most common variants among patients with the neuronopathic form who underwent genetic testing in our cohort, according to previous investigations [20]. We did not find significant differences in the enzyme activity between patients with the two forms, which corresponded with previously published data [17,18].

### 5.1. The Comparison of the First Symptoms Between Neuronopathic and Non-Neuronopathic Forms

Our data suggested that the median age of the first symptoms in neuronopathic patients was 1.0 (0; 2), similar to HOS data 1.5 (0.3, 3.0) years, as well as in neuronopathic patients: 2.0 (1.0; 4.0) vs. 2.0 (0.3, 4.5) years, respectively. Median diagnosis age in our cohort was 3.0 (2; 4) vs. 3.0 (1.4, 5.3) years in the neuronopathic/non-neuronopathic form compared to cumulative HOS data, presented as medians and [10%, 90%]—3.0 [1.4; 5.3] vs. 4.0 [1.0, 7.4] years [5].

First-year psychomotor development in MPS II infants could remain non-delayed and be equal to healthy peers. The following delay of the neurocognitive development may be variable, even in severe (neuronopathic) forms, with stabilization at the age of 4–4½ years, followed by progression of cognitive impairment [21]. Intellectual development in patients with attenuated mucopolysaccharidosis type II (MPS II), a non-neuronopathic form, is comparable to that of healthy peers, although they may exhibit attention deficits and challenges in executive functioning and visual-motor skill acquisition [22].

According to Chinese researchers, it is possible to reliably diagnose a non-neuronopathic form only after the age of 6. By the age of 6, cognitive deficits were detected in 94.3% of patients [6]. However, for ages six and older, there may be inaccuracies in determining the type of MPS 2 [5]. The data from previous studies were used to form the sample in our study: exclusion of patients under the age of 5 and exclusion of patients who did not adhere to the phenotype during the dynamic observation period. For example, three patients with initially mild symptoms over the age of 12 developed intellectual disability: two with mild intellectual disability and one with unspecified intellectual disability. This may be due to complications of the underlying disease or other causes of CNS involvement. Alternatively, it may be due to the unclear distinction between the different forms of MPS II at a younger age, resulting in a delayed onset of CNS symptoms and cognitive deficits. These patients were excluded from further analysis. Similar transitions from non-neuronopathic to neuronopathic forms and vice versa have been observed by other researchers based on data from the HOS registry [5], which also highlights the complexity of differentiating between the different forms of MPS II. If we refer to the literature data, we can see another final age cutoff for dividing patients into severe and attenuated ones in the authors analyzing the HOS register—this was an age of 10 years [5]. This reflects the complexity of differentiating the forms of MPS and possibly the need to distinguish an intermediate phenotype or a neuronopathic phenotype with late manifestation.

### 5.2. Analysis of the Clinical Phenotypes

The clinical characteristics of the main symptoms of MPS correspond to the literature data [2,5,16]. The HOS registry data showed that the most common symptoms of MPS II in any form were facial roughness, joint stiffness, and hepatomegaly, which were reported in >80% of patients [5]. In our study, Hurler phenotype, short neck, stiff large joints, and hepatosplenomegaly were observed at a frequency above 90% in both patient groups.

Retrospective analysis of clinical manifestations by MPS II forms according to the HOS registry: common manifestations in the neuronopathic/non-neuronopathic group: coarse facial features (91.0% vs. 82.4%), joint stiffness and limited function (85.0% vs. 88.2%), hepatomegaly (87.0% vs. 80.4%), hernias (78.0% vs. 74.5%), and valve disease (75.0% vs. 78.4%). According to our data, similar results were obtained for the neuronopathic/non-neuronopathic forms: coarse facial features 81/81 (100%) vs. 38/41 (92.3%), stiffness of large joints 78/81 (96.3%) vs. 35/37 (94.6%), hepatomegaly (as part of splenomegaly): 80/82 (97.6%) vs. 37/41 (90.2%), hernias: 71/77 (92.2%) vs. 33/41 (80.5%), myxomatous valve degeneration 65/79 (82.3%) vs. 23/38 (60.5%).

In our cohort, the frequency of symptoms was high in almost all cases, with the exception of cardiovascular involvement in the non-neuronopathic form, where the frequency of symptoms was lower (Table 5).

### 5.3. Analysis of Orthopedic Manifestations and Arthropathy

According to HOS, orthopedic manifestations are typical for the majority of MPS II patients, regardless of the age of onset and severity of the disease. 90.2% of patients had skeletal deformity [23], similar to our data: 81/81 (100%) patients with the neuronopathic form and 40/41 (97.6%) patients with the non-neuronopathic form. The five most common manifestations, typical for MPS II patients were observed in HOS registry: coarse facial features (198/245; 80.8%), typical claw hand deformity (130/245; 53.1%), kyphosis/gibbus (88/245; 35.9%), scoliosis (45/245; 18.4%), and foot deformity (16/245; 6.5%) [23]. In our group the coarse face (Hurler phenotype) and claw hand was frequently observed in both neuronopathic (100% and 77.6%) and non-neuronopathic (92.3% and 61.3%, respectively) forms of MPS II in our study, spine deformities (curvatures) were more typical for neuronopathic form (56/76; 73.7%), compared to non-neuronopathic form (26/40; 65.0%), chest deformity was equal in both forms—47/81, 58.0% in neuronopathic and 23/40, 57.5% in non-neuronopathic.

Patients with joint stiffness and limited function were more likely to have cardiovascular and pulmonary manifestations, as well as symptoms of central and peripheral nervous system involvement (Fisher’s exact test: *p* ≤ 0.0002) [23] according to the HOS registry data. In our cohort, the neuronopathic phenotype was associated with involvement of the ankle, knee, and small joints of the feet, manifested as joint deformities.

### 5.4. Analysis of Respiratory Disorders

Respiratory disorders are a common symptom in patients with MPS II and reach 90% for all types of MPS [24]. In MPS II, they appear as early as the first year of life [25]. Respiratory disorders are based on ear, nose and throat involvement (otorhinolaryngological manifestations) and obstructive sleep apnea syndrome caused by hypertrophy of the lymphoepithelial pharyngeal ring, neck anatomy features (shortening, restriction of movements in the atlantoaxial joint), macroglossia and laryngeal rigidity, arthropathy of the temporomandibular joint, thickening of soft tissues in the laryngopharynx and tracheomalacia. As a rule, ENT symptoms usually appear before the diagnosis of MPS [24]. In our study, the frequency of respiratory disorders at the time of MPS diagnosis was 56.3% in patients with the neuronopathic form and 34.2% in patients with the non-neuronopathic form of MPS II.

The analysis of the presence of sleep apnea in patients with MPS II is of particular interest. According to our study, this symptom prevailed in the non-neuronopathic form group at the time of diagnosis (median age of neuronopathic/non-neuronopathic form 3.0/7.0 years) and was significantly more common in the same group in the second time-point (median age of neuronopathic/non-neuronopathic form 14 vs. 22 years).

It is possible that these results can be explained by the older age of patients in the non-neuronopathic form group in both the first and second time-points. According to the literature, the incidence of obstructive sleep apnea among patients with MPS can reach 35–71%, regardless of the form of the disease [24,26]. In addition, the incidence of sleep apnea at the onset of the disease can be modified by adenotonsillectomy, and in the later stages of the disease, the frequency of obstructive sleep apnea in patients with MPS increases repeatedly in connection with the development of oropharyngeal stiffness [24,26]. According to previous studies, apnea in the late stages of the disease is a reliable predictor of death [20].

### 5.5. Treatment Analysis

Treatment adherence: According to the literature, 94% of patients with the neuronopathic form and 92.2% of patients with the non-neuronopathic form received ERT [5], and in our study, 69.4% of patients with the neuronopathic form and 30.7% of patients with the non-neuronopathic form received ERT. Adherence to therapy was higher in patients with severe MPS type II. The treatment coverage in Russian patients with MPS type II is lower than according to HOS [5]. The average age at ERT starting in patients with the neuronopathic form was earlier—5.0 (3.0; 8.0) years, compared to patients with non-neuronopathic forms—7.5 (3.0; 21.0) years (*p* = 0.044). According to the HOS registry, ERT started approximately one year later, but the differences in the start of ERT were insignificant in groups with a neuronopathic form of 6.5 (2.9, 10.1) years compared to a non-neuronopathic form of MPS type II patients—6.7 (2.7, 9.2) years [5].

Based on the features we have selected, it is possible to form a portrait of a patient with a neuronopathic form of MPS type II, as well as to identify these symptoms as predictors of a severe course of MPS type II in children under the age of three. Delayed mental and speech development at the age of 1 to 3 years, combined with apnea at the time of diagnosis, as well as heart involvement, especially in the form of myxomatous degeneration of the mitral and aortic valves, will be the most typical manifestations for a patient with a severe form of MPS type II. The presence of epilepsy, feet, ankles, and knee joint stiffness and limited function, impaired swallowing, and inability to walk independently after the age of five years will increase the likelihood of subsequent development of the neuronopathic form in a patient with MPS type II.

Russian literature describes predictors of the neuronopathic form in children aged three years: the absence of control over pelvic organ functions by the age of three, combined with the lack of the ability to construct common sentences, as well as the presence of enlarged brain ventricles according to neurosonography [27].

### 5.6. New Approaches of Enzyme Replacement Therapy

Currently, ERT is the primary form of pathogenetic treatment for MPS II. However, large-molecular-weight molecules—idursulfase and idursulfase beta—are unable to cross the blood–brain barrier, which significantly limits the efficacy of this therapy for treating neuronopathic forms of MPS [28]. In 2021, Japanese researchers published the first data on the successful use of idursulfase conjugated to a transferrin receptor, which enabled the blood–brain barrier to be crossed in an animal model [29]. That same year, the efficacy of pabinafusp alfa in patients with MPS II was demonstrated in Japan in phase 1/2 and 2/3 clinical trials [14,30] and in a group of patients in Brazil (phase 2). Positive changes in neurocognitive parameters were observed following patient testing, alongside a reduction in GAG levels in cerebrospinal fluid, serum, and urine upon intravenous administration of pabinafusp alfa, while maintaining a favorable safety profile [31].

The drug HIR-Fab-IDS—verenafusp alfa, has been developed in the Russian Federation; it consists of a conjugate of the Fab fragment of an antibody against the human insulin receptor and recombinant modified iduronate-2-sulfatase [32]. It is capable of crossing the blood–brain barrier, as demonstrated by interim results from a multicenter, open-label, multi-cohort study evaluating the safety, pharmacokinetics, pharmacodynamics, and efficacy of verenafusp alfa in 18 patients with MPS II [33]. A reduction in heparan sulfate levels—which are implicated in neurodegenerative processes in neuronopathic forms of MPS II—was observed in both cerebrospinal fluid and urine. Improvements and/or stabilization were noted in neurocognitive development, behavior, and the somatic manifestations of the disease with a high safety profile [33].

A recent achievement is the development and clinical implementation of a combined treatment regimen for the neuronopathic forms of MPS II, comprising intrathecal idursulfase administration and intravenous ERT. Idursulfase for intrathecal administration has been developed and has undergone Phase 2/3 clinical trials using the SOPH-A-PORT Mini S implantable access port. The study noted a decrease in GAG levels based on cerebrospinal fluid analysis across all age groups, as well as positive trends in psycho-speech development among children under the age of 6 with missense variants in the *IDS* gene [34]. A study involving a group of 56 children with the neuronopathic form of MPS II served as a continuation of the Phase 2/3 trial [28]. Researchers classified 49/56 (88%) patients as having improved, stabilized, or experienced slowed disease progression. The best outcomes regarding neurocognitive development and skill acquisition were observed in 13 children who began receiving intrathecal idursulfase early and were under the age of 6 at the start of the study. Large rearrangements of the *IDS* gene, various survey data indicated improvement or stabilization of development in 55–67% of cases (10–12 out of 18 individuals) [28].

Intracerebroventricular administration of idursulfase beta is analogous to intrathecal administration via an implanted cerebrospinal fluid reservoir placed in the brain ventricles. Researchers observed improvements in neurocognitive development that correlated with a reduction in heparan sulfate levels in the cerebrospinal fluid. The most favorable outcomes regarding neurocognitive development were observed in the group of children who began therapy at the age of 3 years or younger [7,8].

Despite the safety and efficacy results associated with intrathecal/intraventricular therapy, intravenous ERT utilizing molecules capable of crossing the blood–brain barrier appears preferable, as it obviates the need for neurosurgical port placement, entails a lower risk of neuroinfection, and allows for a single route of drug administration.

Effective application of new approaches to ERT is possible only if the diagnosis of the neuronopathic form of MPS II is established early (before the age of 3–6 years). We hope the early identification of the neuronopathic form will engage the creation of the spectrum of new drugs for neuronopathic forms not only of MPS type II, but also neuronopathic forms of other types of MPS (MPS type III, MPS type VII). Not only optimization of the drug delivery systems and approaches, but design the new form of the drugs, capable of crossing the blood–brain barrier, e.g., enzymes fused with transferrin or insulin receptor [8,33].

### 5.7. Outcomes Analysis

According to the HOS register [5], it was found that patients with severe forms have a higher frequency of death. According to the international authors, there is a greater difference between the frequency of fatal outcomes in non-neuronopathic forms, 1/51 (2%) in the HOS registry and 5/42 (11.9%) in our cohort, with similar death rates—15/100 (15%) in HOS and 13/82 (15.9%) in our cohort patients with neuronopathic forms. This shift towards more deaths in the non-neuronopathic form in the Russian cohort might be explained by a greater median age at the end of the study in the group of neuronopathic form, 15.7 and 17 years according to HOS data, compared to the age of 14 and 22 years in our cohort, and lower treatment adherence in Russian patients with attenuated forms, compared to HOS data. The main causes of death were similar to the international data: the most common causes of death were cardiovascular failure in 5 (non-neuronopathic/neuronopathic forms 3/2) out of 18 cases (27.8%), and respiratory failure in 5 (non-neuronopathic/neuronopathic forms 2/3) cases (27.8%).

### 5.8. Limitations

The retrospective type of registry, missing data, personal opinion of the physician, and the accuracy of the information included in the registry might influence the study results. Different times to diagnosis and start of treatment might influence the study’s outcomes. Our study did not evaluate the confounding variables that could affect outcomes, such as time before the diagnosis and treatment or differences in healthcare access. The absence of the ROC analysis predictive models and positive predictive values and absence of the validation cohort make the ability of these predictors to support actual clinical decision-making remain uncertain. These factors could skew the results and interpretations. A more comprehensive analysis that accounts for these confounders would provide more robust and reliable findings.

## 6. Conclusions

Identifying symptoms characteristic of severe forms of mucopolysaccharidosis and searching for predictors of a severe course of type II mucopolysaccharidosis are important not only for determining the clinical form of the disease but also for developing various approaches to treating such patients. Early diagnosis of severe forms of type II mucopolysaccharidosis will make it possible to use currently approved methods of intraventricular enzyme replacement therapy, which will improve the prognosis for such patients. Symptoms that predict the risk of developing the neuronopathic form can be used in clinical practice.

## Figures and Tables

**Table 1:** Dynamics of psychomotor, speech, and cognitive/mental development in groups of patients with neuronopathic and non-neuronopathic forms of MPS II, stratified by age/according to age.

MPS II Form, *n* (%)	Psychomotor Development up to 1 Year	Mental and Speech Development 1–3 Years	Intellectual Disability 5–12 Years	Intellectual Disability After 12 Years
**Non-neuronopathic form** ***n* = 42 (100)**	Normal, *n* = 39 (92.9)	Normal, *n* = 34	No cognitive decline	
		Delayed, *n* = 5 (11.9)		
	Delayed, *n* = 3 (7.1)	Normal, *n* = 2 (4.8)		
		Delayed, *n* = 1 (2.4)		
**Neuronopathic form, *n* = 82 (100)**	Normal, *n* = 34 (41.5)	Normal, *n* = 2 (2.4)	Mild, *n* = 1 (1.2)	Unspecified, *n* = 1 (1.2)
			Moderate, *n* = 1 (1.2)	Moderate, *n* = 1 (1.2)
		Delayed, *n* = 32 (39.0)	Mild, *n* = 22 (26.8)	Mild, *n* = 6 (7.3)
				Moderate, *n* = 2 (2.4)
				Severe, *n* = 1 (1.2)
			Moderate, *n* = 1 (1.2)	Profound, *n* = 1 (1.2)
			Severe, *n* = 5	Severe, *n* = 3 (3.7)
			Profound, *n* = 2 (2.4)	Profound, *n* = 2 (2.4)
			Unspecified, *n* = 2 (2.4)	Unspecified, *n* = 2 (2.4)
	Delayed, *n* = 48 (58.5)	Normal, *n* = 3 (3.7)	Mild, *n* = 2 (2.4)	Mild, *n* = 2 (2.4)
			Unspecified, *n* = 1 (1.2)	-
		Delayed, *n* = 45 (54.9)	Mild, *n* = 23 (28.0)	Mild, *n* = 4 (4.9)
				Moderate, *n* = 2 (2.4)
				Severe, *n* = 3 (3.7)
				Profound, *n* = 3 (3.7)
				Unspecified, *n* = 1 (1.2)
			Moderate, *n* = 7 (8.5)	Moderate, *n* = 2 (2.4)
				Severe, *n* = 3 (3.7)
				Profound, *n* = 1 (1.2)
			Severe, *n* = 8 (9.8)	Severe, *n* = 4 (4.9)
				Profound, *n* = 1 (1.2)
			Profound, *n* = 4 (4.9)	Profound, *n* = 3 (3.7)
			Unspecified, *n* = 3 (3.7)	Unspecified, *n* = 1 (1.2)

**Table 2:** Comparative characteristics of symptoms of mucopolysaccharidosis with neuronopathic and non-neuronopathic forms.

Parameter	Parameter	NPF, *n*, Me (Q1; Q3) *n* = 82	Non-NPF, *n*, Me (Q1; Q3) *n* = 42	*p*-Value
Severe *IDS* genetic variants (pathogenic missense mutations + gross rearrangements)		30/46 (65.2)	16/46 (34.8)	0.004
Pathogenic missense mutations		18/57 (31.6)	15/57 (26.3)	0.536
Gross rearrangements		12/63 (19.0)	1/38 (2.6)	0.018
Age of death, years min-max	Age of death, years min-max	14.0 (12; 20) 9–22	21.0 (21.0; 41.0) 18.0–43.0	0.015
Age of first symptom, years min-max	Age of first symptom, years min-max	1.0 (0; 2) 0–5	2.0 (1.0; 5.0) 0–19	0.008
Age at diagnosis, years min-max	Age at diagnosis, years min-max	3.0 (2; 5) 0–27	7.0 (2.0; 12.0) 0–38	0.002
Age of start ERT, years min-max	Age of start ERT, years min-max	5.0 (4.0; 8.0), 1–42	10.0 (4.0; 22.0) 2.0–43.0	0.010
Age at last observation min-max	Age at last observation min-max	14.0 (11.0; 18.0) 6.0–47.0	22.0 (13.0; 31.0) 6.0–50.0	0.0006
Ever received ERT, *n* (%)	Ever received ERT, *n* (%)	77/81 (97.1)	32/42 (76.2)	0.002
Normal psychomotor development up to 1 year, *n* (%)	Normal psychomotor development up to 1 year, *n* (%)	34/82 (41.5)	39/42 (92.9)	0.0000001
Psychomotor development delay (up to 1 year), *n* (%)	Psychomotor development delay (up to 1 year), *n* (%)	48/82 (58.5)	3/42 (7.1)	0.0000001
Delayed mental and speech development (1–3 years), *n* (%)	Delayed mental and speech development (1–3 years), *n* (%)	77/82 (93.9)	6/42 (14.3)	0.0000001
Intellectual disability: from 5 to 12 years), *n* (%)	No	0/82 (0)	42/42 (100.0)	0.0000001
Intellectual disability: from 5 to 12 years), *n* (%)	Mild	48/82 (58.5)	na	0.0000001
Intellectual disability: from 5 to 12 years), *n* (%)	Moderate	9/82 (11)	na	0.0000001
Intellectual disability: from 5 to 12 years), *n* (%)	Severe	13/82 (15.9)	na	0.0000001
Intellectual disability: from 5 to 12 years), *n* (%)	Profound	6/82 (7.3)	na	0.0000001
Intellectual disability: from 5 to 12 years), *n* (%)	Unspecified	6/82 (7.3)	na	0.0000001
Intellectual disability after 12 years, *n* (%)	No	0/48 (0)	34/34 (100.0)	0.0000001
Intellectual disability after 12 years, *n* (%)	Mild	11/48 (22.9)	na	0.0000001
Intellectual disability after 12 years, *n* (%)	Moderate	7/48 (14.6)	na	0.0000001
Intellectual disability after 12 years, *n* (%)	Severe	14/48 (29.2)	na	0.0000001
Intellectual disability after 12 years, *n* (%)	Profound	12/48 (25.0)	na	0.0000001
Intellectual disability after 12 years, *n* (%)	Unspecified	4/48 (8.3)	na	0.0000001
Hurler phenotype, *n* (%)	Hurler phenotype, *n* (%)	81/81 (100)	38/41 (92.3)	0.014
Hydrocephalus, *n* (%)	Hydrocephalus, *n* (%)	45/78 (57.7)	11/38 (29.0)	0.004
Epilepsy, *n* (%)	Epilepsy, *n* (%)	19/79 (24.1)	1/39 (2.6)	0.003
Swallowing disorder, *n* (%)	Swallowing disorder, *n* (%)	37/78 (47.4)	0/35 (0)	0.000001
Hearing loss, *n* (%)	Hearing loss, *n* (%)	59/74 (79.7)	24/38 (63.2)	0.058
Independent walking failure (5–12 years), *n* (%)	Independent walking failure (5–12 years), *n* (%)	12/77 (16.9)	0/42 (0)	0.007
Independent walking failure (>12 years), *n* (%)	Independent walking failure (>12 years), *n* (%)	22/48 (45.8)	1/32 (3.1)	0.0001
Cardiac disorders, *n* (%)	Cardiac disorders, *n* (%)	70/79 (88.6)	27/39 (69.2)	0.010
Cardiomyopathy, *n* (%)	Cardiomyopathy, *n* (%)	32/80 (40.0)	8/38 (21.1)	0.042
Myxomatous valve disease, *n* (%)	Myxomatous valve disease, *n* (%)	65/79 (82.3)	23/38 (60.5)	0.011
Noisy breathing at the time of diagnosis, *n* (%)	Noisy breathing at the time of diagnosis, *n* (%)	31/64 (48.4)	18/35 (41.5)	0.776
Noisy breathing at the last visit, *n* (%)	Noisy breathing at the last visit, *n* (%)	24/61 (39.3)	15/28 (53.6)	0.209
Apnea at the time of diagnosis, *n* (%)	Apnea at the time of diagnosis, *n* (%)	12/40 (30.0)	10/26 (38.5)	0.476
Foot deformity, *n* (%)	Foot deformity, *n* (%)	30/56 (53.6)	6/30 (20.0)	0.003
Foot contracture, *n* (%)	Foot contracture, *n* (%)	29/55 (52.7)	7/30 (23.3)	0.009
Ankle deformity, *n* (%)	Ankle deformity, *n* (%)	33/56 (58.9)	8/31 (25.8)	0.003
Knee deformity, *n* (%)	Knee deformity, *n* (%)	34/56 (60.7)	12/31 (38.7)	0.048
Knee pain, *n* (%)	Knee pain, *n* (%)	8/51	0/31	0.029

**Abbreviations:** ERT—enzyme replacement therapy, Me—median, Q—quartile, na—not applicable, NPF—neuronopathic form, non-NPF—non-neuronopathic form.

**Table 3:** Predictors of the following development of neuronopathic forms of MPS type II.

MPS Type II Features	*Se*	*Sp*	OR (95% CI)	*p*-Value
Severe *IDS* genetic variants	65.2	65.2	3.5 (1.8; 8.3)	0.004
Gross rearrangements	19.0	97.4	8.7 (1.1; 69.9)	0.019
Ever received ERT	95.1	23.8	6.0 (1.8; 20.6)	0.0018
Psychomotor development delay (up to 1 year) *	58.5	92.9	18.4 (5.2; 64.3)	0.0000001
Delayed mental and speech development (1–3 years) *	93.9	85.7	92.4 (26.4; 322.9)	0.0000001
Hydrocephalus *	80.4	45.0	3.3 (1.5; 7.7)	0.004
Epilepsy *	95	38.8	12.0 (1.5; 93.6)	0.003
Hearing loss	79.7	36.8	2.3 (1.0;5.5)	0.058
Swallowing disorder	47.4	100	-	0.000001
Independent walking failure (5–12 years)	15.6	100	-	0.007
Independent walking failure (after 12 years)	45.8	96.9	26.2 (3.3; 208.0)	0.0001
Cardiac disorders *	88.6	30.8	3.5 (1.3; 9.1)	0.010
Myxomatous valve disease	73.9	51.7	3.0 (1.3; 7.2)	0.011
Foot deformity	53.6	80.0	4.6 (1.6; 13.0)	0.003
Foot contracture	52.7	76.7	3.7 (1.4; 9.9)	0.009
Ankle deformity	58.9	74.2	4.1 (1.6; 10.8)	0.003
Knee deformity	60.7	61.3	2.4 (1.0;6.0)	0.048

* The most meaningful predictors are bolded. Abbreviations: ERT—enzyme replacement therapy.

**Table 4:** Multivariate regression analysis of the early predictors of the following development of neuronopathic forms of MPS type II.

	b	Std. Err. of b	t	*p*-Value
Intercept	0.011	0.067	0.17	0.867
Psychomotor development delay (up to 1 year), yes	0.192	0.057	3.39	0.001
Delayed mental and speech development (1–3 years), yes	0.692	0.060	11.52	0.0000001
Cardiac disorders, yes	0.129	0.067	1.92	0.058

**Table 5:** Comparative characteristics of the main symptoms of MPS according to HOS and Russian register data.

Symptom	Neuronopathic Form	Neuronopathic Form	Non-Neuronopathic Form	Non-Neuronopathic Form
Symptom	HOS data	Our data	HOS data	Our data
Coarse facial features (%)	91.0	100	82.4	92.3
Joint stiffness and limited function (%)	85.0	96.3	88.2	94.6
Hepatomegaly (%)	87.0	97.6	80.4	90.2
Hernias (%)	78.0	92.2	74.5	80.5
Cardiovascular features (%)	83.0	88.6	82.4	69.2
Valve disease (%)	75.0	82.3	78.4	60.5

Abbreviations: HOS, Hunter Outcome Survey.

## Data Availability

The data presented in this study are available on request from the corresponding author, because the data are not publicly available due to privacy or ethical restrictions.

## Abbreviations

CNS	central nervous system
GAGs	glycosaminoglycans
ERT	enzyme replacement therapy
HOS	Hunter Outcome Survey
IDS	iduronate-2-sulfatase
MPS II	mucopolysaccharidosis type II
